# Twenty years of health monitoring in a conventional neuroscience animal facility: challenges, strategies, and 3Rs-oriented approaches to animal welfare, personnel health, and research integrity

**DOI:** 10.3389/fphys.2026.1821583

**Published:** 2026-07-15

**Authors:** Daniele Peluso, Paola Bonsi, Cecilia Gentile, Martina Montanari, Cesidio Romano, Annarita Wirz

**Affiliations:** 1 PhD School of Applied Medical-Surgical Sciences, University of Rome “Tor Vergata”, Rome, Italy; 2 Department of Biology, University of Rome “Tor Vergata”, Rome, Italy; 3 Department of Experimental Neuroscience and Models of Neurological Diseases, LIFE - Istituto di Ricerca e Cura - Santa Lucia IRCCS, Rome, Italy; 4 Department of Medicine and Surgery, Università Campus Bio-Medico di Roma, Rome, Italy; 5 Plaisant, Tecnopolo Roma, Rome, Italy

**Keywords:** 3Rs, culture of care, pathogen surveillance, refinement, rodents

## Abstract

Rodent health-monitoring programmes are essential for maintaining colony health, supporting health-related aspects of animal welfare and personnel biosafety, and providing relevant contextual information for the interpretation of experimental data. Subclinical infections may remain unnoticed while influencing animal physiology, experimental variability, and colony-management decisions. However, long-term retrospective descriptions of pathogen trends and programme-level changes in conventional animal facilities remain limited. This retrospective study aimed to describe long-term trends in microbiological findings in a conventional rodent facility, identify recurrent or emerging pathogens, and explore how observed changes over time coincided with programmatic modifications in housing, husbandry, hygiene, personnel training, and diagnostic procedures. We performed a retrospective analysis of 20 years of health monitoring (March 2006–December 2025) in a conventional neuroscience facility housing mice and rats. The dataset comprised 135, 558 diagnostic assays (pathogen-specific test records). Positivity to pathogens was defined as positives/(positives+negatives), with 95% Wilson confidence intervals. Temporal trends were tested using binomial logistic regression on yearly aggregated counts (odds ratio per year), with pathogen-level p-values controlled by Benjamini–Hochberg FDR. Genotype comparisons were restricted to binary batches (Genetically Modified vs Non-Genetically Modified), and seasonality to predefined surveillance months. Overall positivity for the presence of pathogens showed a significant decrease over years in both species (p<0.001). Genotype effects were significant in mice (p<0.001) but not in rats (p=0.144). Seasonality was significant in mice (p<0.001) but not in rats (p=0.24). Continuous health monitoring highlighted species and subgroup-specific patterns relevant to risk assessment and colony management. Over two decades, progressive and structured improvements in health-surveillance practices, together with adaptive management procedures and qualified staff training, coincided with a sustained decrease in pathogen positivity. These improvements support a welfare-oriented and progressive refinement- informed approach embedded in an institutional commitment to animal health, personnel well-being, biosafety, transparent communication, and responsible colony management.

## 1 Introduction

Health Monitoring (HM) of laboratory rodent colonies represents a cornerstone in the management of modern animal facilities, with direct implications for animal health, personnel safety, and the interpretation of experimental data. The presence of infectious agents can alter physiological, immunological, and behavioural parameters, introducing confounding variables affecting the interpretation of experimental results (Buchheister and Bleich, 2021).

Retrospective studies conducted in conventional animal facilities have documented a high incidence of bacterial, viral, and parasitic agents in apparently healthy colonies, highlighting how microbiological contamination represents a structural rather than episodic critical issue (Carriquiriborde et al., 2020). Even frequently asymptomatic pathogens have been associated with interference in experimental parameters despite the absence of evident clinical signs (Drake et al., 2008), confirming that apparent phenotypic normality does not necessarily coincide with physiological stability. In this context, maintaining a known and controlled health status of colonies constitutes an essential prerequisite for ensuring the reliability and validity of biomedical studies (Carriquiriborde et al., 2020).

Over the past decades, the role of health monitoring has progressively evolved. This evolution reflects a shift from a paradigm centered on maximal pathogen exclusion and prevention of clinical disease to a more articulated model focused on controlling biological variables and managing the microbiological complexity of colonies (Buchheister and Bleich, 2021). From this perspective, health monitoring is no longer viewed solely as a biosafety tool, but as an integral component of methodological quality and physiological robustness of experimental models. This evolution has been formalized in the recommendations of the Federation of European Laboratory Animal Science Associations (FELASA), which define shared criteria for the design of health monitoring programs, identifying agents to be monitored, sampling frequencies, and the most appropriate diagnostic methodologies according to the species housed, the type of facility, and research purposes (Mähler et al., 2014).

In the European context, Directive 2010/63/EU formally recognized the importance of health monitoring [Annex III, Section 3.1(a)] and laboratory animal welfare [Art. 33.1(a)], and established the principle of the 3Rs (Replacement, Reduction, and Refinement) as the foundation of responsible animal use for scientific purposes. Building on the original framework proposed by Russell and Burch (1959), the 3Rs are now understood as an integrated ethical and scientific approach aimed at replacing animal use whenever valid non-animal methods are available, reducing the number of animals used through rigorous experimental design and analysis, and refining all aspects of animal care and procedures to minimise pain, suffering, distress or lasting harm while improving animal welfare and scientific quality. In the context of the present retrospective analyses, however, the 3Rs are used as a conceptual and regulatory framework to support the study. Within this regulatory framework, regular health monitoring of colonies represents an explicit requirement of animal health management and microbiological surveillance. When integrated with documented reporting, veterinary oversight, corrective actions, and periodic review of husbandry and housing practices, health monitoring may support Refinement-related decision-making by contributing to animal physical health, health-related welfare, and the continuous improvement of colony management practices.

The various infectious agents that may affect laboratory rodent colonies have different effects on life, health, or scientific quality. Some pathogens, such as highly pathogenic viruses (e.g., *Sendai virus* or *Lymphocytic choriomeningitis virus*) and bacteria such as *Clostridium piliforme*, are capable of causing acute disease or mortality, especially in large cohorts. Other opportunistic microorganisms, including *Rodentibacter spp*, *Helicobacter spp*, and *Mycoplasma pulmonis*, may cause clinical or subclinical infections in susceptible strains or immunodeficient animals. Finally, frequently asymptomatic agents such as *murine parvoviruses*, although not causing evident clinical signs, may substantially influence experimental outcomes, particularly in fields such as immunology, inflammation, and oncology (Buchheister and Bleich, 2021; Simmons et al., 2024).

To address these microbiological threats, HM programs have historically relied on sentinel animals exposed to soiled bedding and periodic virological, bacteriological, and parasitological testing. The design of these programs, including the definition of sampling frequency and the number of animals involved, can impact diagnostic effectiveness and colony safety. However, the evolution of knowledge and diagnostic technologies has progressively highlighted the limitations of exclusively periodic monitoring based on dedicated animals, both in terms of diagnostic sensitivity and animal welfare impact. Indeed, programs based on sentinel animals exposed to soiled bedding may show variable sensitivity depending on the pathogen and its transmission dynamics within the colony, with possible delays in the detection of subclinical infections (de Bruin et al., 2016; Simmons et al., 2024).

In recent years, alternative approaches to soiled bedding sentinels (SBS) based on environmental health monitoring strategies (EHM) including Exhaust Dust Testing (EDT) and sentinel free soiled bedding (SFSB) techniques have demonstrated improved pathogen detection capacity, with sensitivity from comparable to 20% higher than traditional programs (LaFollette et al., 2024). At the same time, EHM allows for a significant reduction or elimination of sentinel animal use, representing a concrete application of the principles of Replacement and Reduction, as well as Refinement, since they combine greater scientific effectiveness with a decrease in invasive procedures and in the overall number of animals used (LaFollette et al., 2024; Simmons et al., 2024; Tu-Wood et al., 2025).

The principle of the 3Rs (Replacement, Reduction, and Refinement) constitutes the ethical and scientific foundation of responsible animal use in biomedical research. Although the three components are formally considered equally important, recent work has highlighted that, in practice, Refinement is often underrepresented compared to Replacement and Reduction, both in terms of funding and academic recognition (Grimm et al., 2023). Refinement measures may sometimes be perceived as ancillary to the data-generation process rather than as elements that can support both animal welfare and scientific reliability (Grimm et al., 2023). In the context of rodent colony management, health monitoring should primarily be regarded as an operational tool for assessing and managing colony health risks. Its contribution to Refinement is therefore indirect, but relevant: by enabling the early detection and management of infectious agents, regular health monitoring can support health-related welfare, guide targeted interventions, and contribute to the progressive improvement of housing, handling, and procedural conditions when specific risks are identified (Grimm et al., 2023).

Within this framework, regular and documented health monitoring may be interpreted as one operational component of evidence-based colony management. The 3Rs and Culture of Care principles may guide how health-monitoring information is collected, interpreted, communicated, and translated into risk-based decisions, staff training, biosafety measures, transparent communication, and targeted management actions. This is particularly relevant in conventional facilities, where less stringent hygienic barriers than in SPF facilities, high cage numbers, multiple experimental protocols, and exchanges of animals with other institutions may increase the risk of pathogen introduction and spread. Under these conditions, a health-monitoring programme tailored to the facility characteristics and consistent with FELASA recommendations can support colony health management, health-related welfare, personnel biosafety, and research-data interpretation.

The aim of the present retrospective single-facility study was to describe long-term trends in pathogen positivity in mouse and rat colonies housed in a conventional neuroscience animal facility over an approximately 20-year period, from March 2006 to December 2025. Specifically, we analysed longitudinal health-monitoring records to characterize changes in the detection of selected pathogens over time, in relation to sampling frequency, diagnostic approaches, and progressive adaptations of the monitoring programme according to FELASA recommendations and facility-specific needs.

We also examined pathogen-positivity patterns according to selected colony-related variables, including animal species, genotype status, and sex, with the aim of identifying differences in detection patterns and risk profiles within the monitored colonies. These comparisons were not intended to establish causal relationships or intrinsic biological susceptibility.

Overall, this study provides a facility-based description of how repeated health monitoring can inform evidence-based colony management in a conventional setting.

## 2 Materials and methods

### 2.1 Study design and dataset construction

A retrospective longitudinal analysis was conducted, based on the health monitoring program active at the animal facility. The observation period covered twenty years (March 2006 to December 2025). The collected data enabled the construction of a structured dataset comprising 135, 558 diagnostic records deriving from health monitoring cycles performed on a quarterly or annual basis, in accordance with FELASA guidelines (Mähler et al., 2014).

Each record included the following information:

- Species (*Mus musculus*, *Rattus norvegicus*)
- Colony type (Genetically Modified, Non-Genetically Modified)
- Pathogen category investigated (viruses, bacteria, mycoplasma, fungi, endoparasites, ectoparasites)
- Test result (pathogen-positive or negative)
- Monitoring frequency (quarterly, annual)
- Reference time period

### 2.2 Health monitoring parameters

#### 2.2.1 Structural context and colony characteristics

The longitudinal HM data have been collected from a single, centralised, conventional animal facility housing daily from a minimum of 1, 000 to a maximum of 2, 300 cages in the considered 20-years timeframe. The conventional animal facility is located on level -1 and is organised into three main functional areas: an animal housing area comprising 14 rooms, with a total surface area of approximately 320 m²; a service area of approximately 200 m²; and experimental laboratories, covering approximately 600 m², where procedures such as surgery, behavioural testing, and organ and tissue collection are performed. The positioning of all animal housing and experimental procedure areas on level -1, separated from other building areas including offices, research laboratories, and common areas, provides several operational and biosafety advantages. This layout supports the efficient organisation of animal care and *in vivo* experimental activities.

Data were derived from mouse and rat colonies, including inbred and outbred strains, and genetically modified lines, including transgenic and knockout animals. During the 20 years considered, the weekly mean number of resident rats ranged from a minimum of 400 to a maximum of 600 animals, whereas the number of mice ranged from 4, 000 to 8, 000. Genetically modified lines were managed through dedicated breeding programs designed to ensure genetic and phenotypic stability and to limit the production of *surplus* animals, as defined in the individual experimental protocols authorized by the competent authorities in accordance with the principle of Reduction and Directive 63/2010 UE.

Animals were housed in conventional cages equipped with filter tops (Tecniplast, model 1264 for mice and model 1291 for rats). Since 2005, rodents were provided with nesting paper; then, from late 2007 environmental enrichment was gradually implemented, and by October 2008, all the cages were provided with nesting houses or tunnels and nesting material (Tecniplast mouse house™ for mice, custom-made methacrylate/acrylic tunnels for rats, and paper nesting material for both species), placed on conventional racks (Tecniplast). Cage changes were performed twice weekly according to standardized procedures. Environmental parameters (12:12 light–dark cycle, temperature 22 ± 2 °C, relative humidity 45–55%) were continuously monitored. Facility infrastructure and equipment were subjected to routine maintenance throughout the entire study period. These conditions were maintained over time, allowing longitudinal comparability of the data.

Environmental enrichment is reported here as part of the housing and husbandry context in which colony and sentinel animals were maintained during the retrospective period analysed. It was not part of the diagnostic health-monitoring procedure and was not used for pathogen detection. However, as a welfare-oriented husbandry measure, enrichment may support species-appropriate behaviour, reduce stress-related discomfort, and contribute to the general health condition of the animals, although these effects were not directly assessed in the present study.

#### 2.2.2 Evolution of management interventions during the study period

Over the analysed twenty-year period, several progressive organizational and structural interventions were introduced as part of facility management and housing/husbandry improvement. These represent important contextual variables for interpreting longitudinal health-monitoring trends:

- In 2007, a structured and accredited training program in laboratory animal science was initiated for technical and research staff. The program focused on proper animal use, application of European and national legislation, implementation of the 3Rs principles, and biosafety. This continuing education program remains active.
- In 2008, environmental enrichment was expanded. In addition to nesting material, nesting houses for mice and tunnels for rats were introduced, with the aim of refinement and stress reduction.
- In 2015, a biometric fingerprint-based access replaced the previous badge-based control system. This change improved entry traceability and restricted facility access to a limited number of authorized researchers per experimental group, in addition to dedicated technical staff, to reduce the risk of pathogen introduction and contribute to maintaining colony health status over time.
- Between 2020 and 2022, the number of rooms dedicated to rats was increased from one to five, improving the physical separation of colonies.
- In 2022, standard cleaning and disinfection procedures were supplemented with periodic environmental steam sanitization.

These modifications were considered in the interpretation of longitudinal dataset trends.

#### 2.2.3 Health monitoring program and use of sentinel animals

Sentinel animals of both sexes were used for health monitoring and were maintained under exposure conditions for at least three months. During the study period, the facility used conventional racks and conventional cages. Each rack had one dedicated sentinel cage, positioned at the bottom of the rack. Colony cages were equipped with filter-covered lids, whereas sentinel cages were maintained as non-filtered cages to facilitate exposure to environmental and bedding-derived material.

At each cage change, a mixed sample of soiled bedding collected from the other cages housed on the same rack was added to the corresponding sentinel cage. The amount of soiled bedding added was approximately 10% of the bedding volume. This procedure was performed at every cage change, usually once or twice per week, depending on the housing room and on management- or research-related requirements. Sentinel animals also received food and water originating from the same monitored colonies when applicable, in order to increase exposure to potential infectious agents circulating within the rack.

Whenever possible, experimental animals at the end of procedures were also included in the health monitoring program and hence in the dataset. This approach was adopted because experimental animals may enter shared laboratory spaces for surgical procedures, behavioural testing, and other activities conducted by multiple operators. Their inclusion was intended to increase the representativeness of the monitoring program without requiring additional dedicated sentinel animals.

At the end of the exposure period or experimental procedures, animals included in the monitoring program underwent health analyses according to the standard protocols of the external diagnostic laboratory to which samples were submitted. Monitoring included serological, bacteriological, mycological, and parasitological analyses, with pathogens classified into quarterly or annual testing panels based on biological risk, in accordance with FELASA recommendations (Mähler et al., 2014).

The specific pathogens included in the health-monitoring panels are listed in **Supplementary Table 1**, grouped by pathogen category, species, monitoring frequency, and diagnostic method. Panels were defined according to FELASA recommendations, biological risk, species housed, and facility-specific monitoring requirements.

Sentinel animals were included in the routine clinical and behavioural observations performed by trained technical staff and were regularly examined by the designated veterinarian, as described in Section 2.2.4. According to routine facility records and clinical/behavioural observations, no overt clinical or behavioural concerns attributable to the addition of soiled bedding were recorded, including abnormal behaviour, increased aggression, or stereotypies, in either female or male sentinel cages.

Environmental HM strategies that replace the need for live sentinel animals (such as exhaust dust testing or sentinel-free soiled bedding testing) were not implemented during the retrospective period analysed in this study. However, as part of the strong institutional commitment to Culture of Care and the 3Rs, strategies to replace live sentinel animals are being planned for the years to come.

#### 2.2.4 Clinical monitoring of animals

Clinical and behavioural observations of animals were performed throughout the study period through daily checks by animal facility technical staff, twice-weekly evaluations by the designated veterinarian, and periodic assessments by researchers according to the timelines specified in their experimental protocols. According to the European Commission recommendations, a set of overarching observational categories was applied to all animal care programmes consisted in Appearance (body, fur, skin, eyes and mouth), Body functions (respiration, food and water intake), Environment (litter, nesting and enrichment materials), Behaviours (social behaviour, posture and mobility, undesirable behaviours like stereotypies and barbering) (European Commission: Directorate-General for Environment, 2018).

Researchers also implemented protocol-specific clinical scoring sheets to document clinical signs, behavioural alterations, or conditions of distress, taking into account the strain studied and the type of research conducted.

#### 2.2.5 Pharmacological interventions for pathogen control

Pharmacological treatments were selectively implemented in defined groups of mice and rats housed in the facility, given the impracticality of treating all colonies simultaneously, after careful considerations in order to rule out possible impacts on research results. The primary goal was to contain pathogen spread, reduce environmental contamination, and improve the overall health status of both treated and untreated animals within the facility. In the presence of bacterial pathogens (including *Klebsiella spp*, *Streptococcus spp*, *Staphylococcus* spp., *Pasteurella spp*, and *Proteus* spp), animals from affected colonies were treated with enrofloxacin administered in drinking water for two consecutive weeks. One week after treatment completion, follow-up microbiological monitoring was performed. Depending on the pathogen investigated, analyzed samples included oropharyngeal swabs, urine swabs, and/or freshly collected fecal pellets. Analyses were conducted according to the standard microbiological procedures routinely adopted by the facility. In cases of intestinal protozoan infections (including *Chilomastix spp*, *Entamoeba spp*, *Trichomonas spp*, and *Giardia* spp), metronidazole was administered in drinking water for 5–7 consecutive days. Five days after treatment completion, parasitological evaluation was performed through fecal pellet examination to verify treatment efficacy. For infestations with *Myobia musculinus*, ivermectin was administered in drinking water for one week. Alternatively, permethrin treatment was performed via indirect exposure by placing permethrin-impregnated cotton material inside the cage, which animals used for nest building, for a minimum period of two weeks. In both cases, one week after treatment completion, follow-up testing was performed using skin scraping and/or adhesive tape (scotch test) to detect residual ectoparasites.

### 2.3 Statistical analysis

#### 2.3.1 Dataset structure and unit of analysis

Health monitoring records collected between March 2006 and December 2025 were included in the statistical analysis. In this study, a monitored cage refers to each individual cage included in a health-monitoring batch. The statistical unit of analysis was the pathogen-specific diagnostic assay record associated with monitored cages and the diagnostic panel applied. For each year and species, aggregated counts included the number of positive results (Positive_n), and the corresponding yearly positivity rate were calculated: Positivity rate = Positive_n/(Positive_n + Negative_n).

#### 2.3.2 Study outcomes

The primary outcome was the overall yearly positivity rate stratified by species.

Secondary outcomes included:

- Yearly positivity rates by pathogen category (bacteria, mycoplasma, parasites, viruses)
- Yearly positivity rates for individual pathogens
- Comparisons by genotype
- Evaluation of seasonal patterns

#### 2.3.3 Statistical analysis

Yearly rates of positivity for the presence of pathogens were calculated as the proportion of positive diagnostic outcomes over the total number of tests performed, and 95% confidence intervals were estimated using the Wilson method. Temporal trends were assessed using binomial logistic regression models applied to yearly aggregated counts. The odds ratio (OR) per year was estimated to quantify the multiplicative change in the odds of pathogen positivity associated with a one-year increase over the study period. For analyses conducted at the individual pathogen level, p-values were adjusted for multiple comparisons using the Benjamini–Hochberg false discovery rate (FDR) procedure. Sex-based comparisons were restricted to monitoring batches containing animals of a single sex to prevent misclassification bias. Similarly, genotype comparisons were limited to batches including exclusively GM or exclusively non-GM animals. Seasonality analyses were confined to the months routinely designated for surveillance (March, June, September, and December) in order to minimize potential confounding due to irregular sampling schedules.

All analyses were performed in R (version 4.5.1, 2025-06-13). Figures were produced using ggplot2; binomial models were fitted using the glm function; confidence intervals were computed with the Wilson method; and multiple testing was controlled using the Benjamini–Hochberg procedure.

## 3 Results

Between 2006 and 2025, a total of 135, 558 diagnostic assays were performed as part of the routine health monitoring program of a conventional animal facility. Here, a diagnostic assay refers to a pathogen-specific test record; therefore, the total number of assays reflects both the number of monitored cages included in each health-monitoring batch and the breadth of the pathogen-specific diagnostic panel applied. The investigations included parasitological, bacteriological, and virological analyses. Of these, 115, 256 assays were conducted on *Mus musculus* (including both genetically modified, GM, and non-genetically modified, non-GM, strains), and 20, 302 assays on *Rattus norvegicus* (both GM and non-GM strains). Routine monitoring was scheduled quarterly (March, June, September, and December), in accordance with FELASA recommendations.

**Figure 1:**
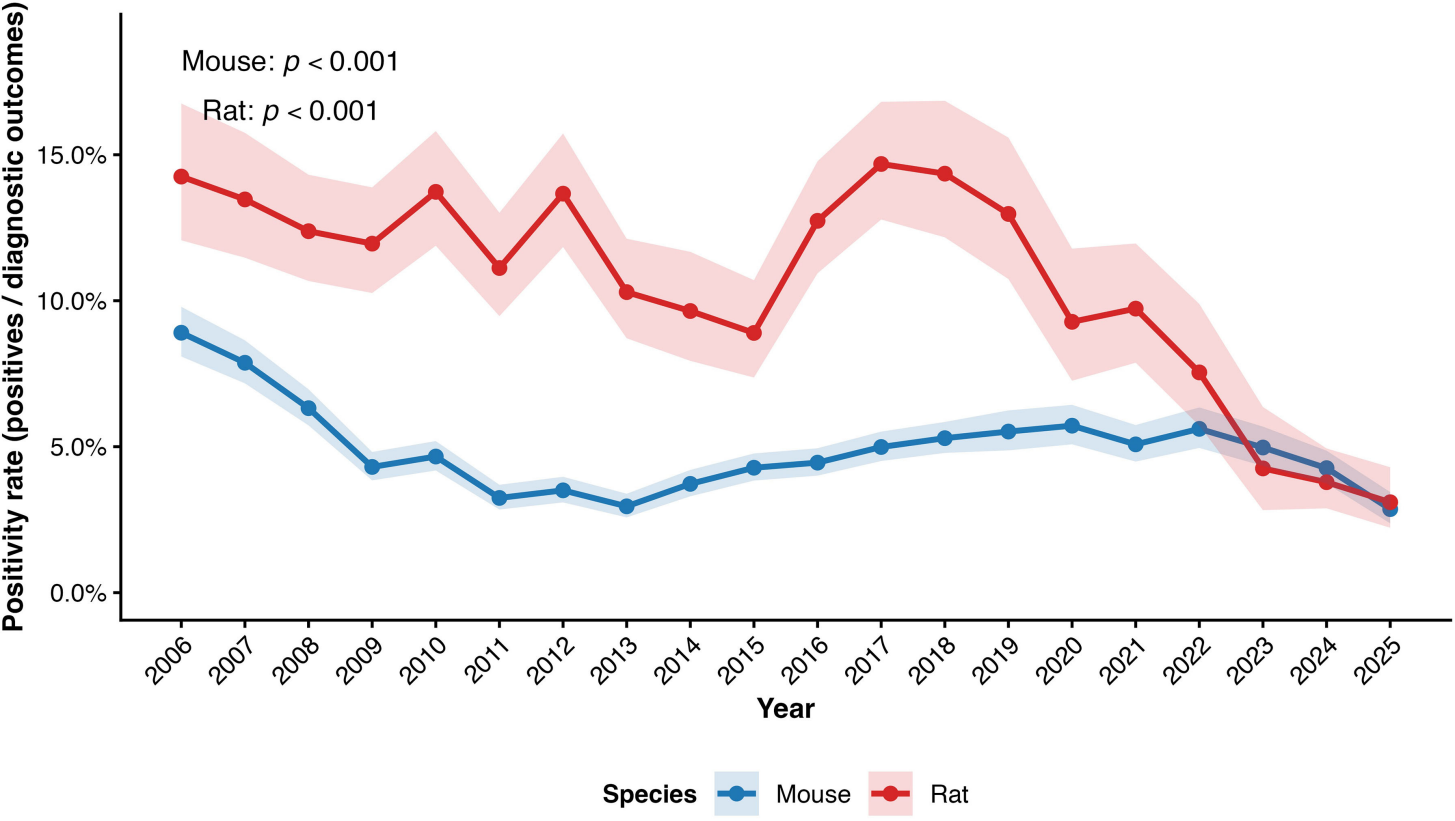
Annual trends of the pathogen positivity rate stratified by species. The graph shows the yearly positivity rate with 95% Wilson confidence intervals.

The longitudinal analysis of data revealed significant temporal variations for several monitored pathogens, as detailed below. **Figure 1** illustrates the trends in pathogen positivity rates over the 20-year study period in both species. Notably, in rats, the positivity rate was consistently higher than in mice for most of the observation period, with initial values around 14–15% in 2006, followed by a gradual decline until 2015. Subsequently, an increase was observed, with a peak between 2017 and 2018 (≈15%), followed by a marked decrease from 2019 onward, reaching values below 5% in the final years of the study (2023–2025). In mice, the positivity rate showed an initial decrease from 2006 to 2011 (from approximately 9% to 3–4%), followed by a progressive increase until 2020–2022, when the highest values were recorded (~5–6%). In the most recent years, a slight decline was observed, with values approaching 3% in 2025. Trend analysis demonstrated a statistically significant reduction over time in the overall positivity rate for both species (p < 0.001).

**Figure 2:**
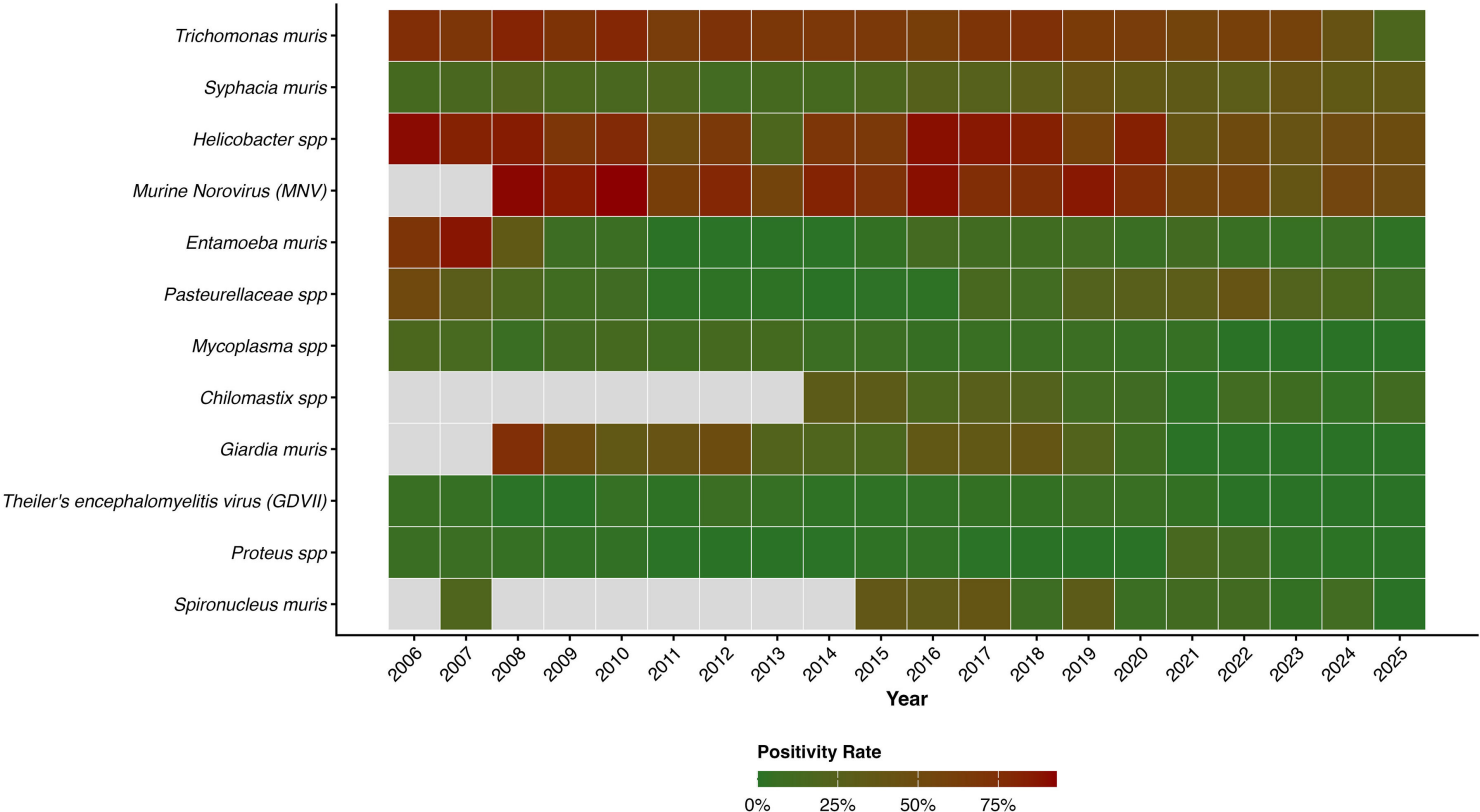
Temporal trend of positivity rates for the main pathogens monitored. The heatmap shows the yearly cumulative positivity rates for relevant pathogens. Grey tiles indicate the absence of detections.

Based on the observed positivity patterns, we further analyzed the data according to pathogen type. **Figure 2** shows the temporal trends in positivity rates for the main monitored pathogens, highlighting a significant variation over time (adjusted annual trend: p < 0.001). Overall, a progressive reduction in positivity was observed for several enteric and bacterial agents, including *Helicobacter spp*, *Murine Norovirus, Giardia muris, Mycoplasma spp*, and *Pasteurellaceae spp*, shifting from moderate-to-high frequencies in the early years to consistently low values in the last five years. *Trichomonas muris* and *Syphacia muris* also showed a decreasing trend, although with persistence at intermediate levels during certain periods. Conversely, pathogens such as *Theiler’s encephalomyelitis virus (GDVII)* and *Proteus spp* exhibited consistently low positivity rates throughout the entire study period. For some agents (e.g. *Chilomastix* spp and *Spironucleus muris*), no detections were recorded in certain years, as indicated by the grey cells in the figure. Overall, the data indicate a progressive improvement in the health status of the monitored colonies over the study period.

**Figure 3:**
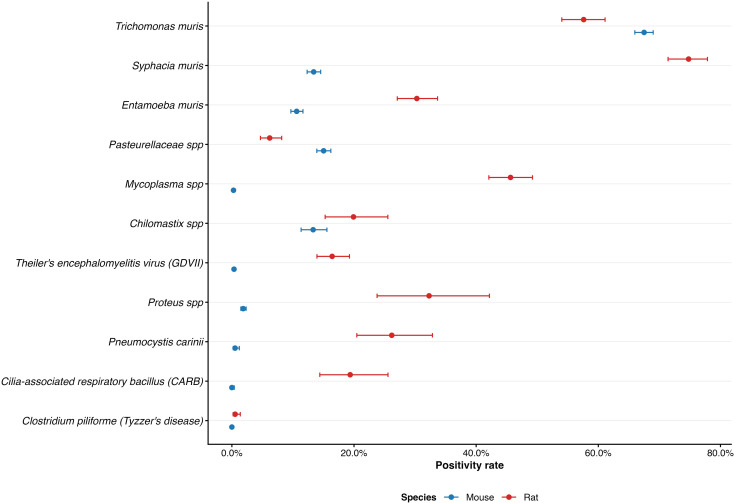
Species-specific sensitivity to pathogens. The graph presents the species-specific positivity rate aggregated over 2006–2025 for pathogens showing a statistically significant difference between mice and rats (p < 0.05), expressed as the proportion of positive samples with 95% confidence intervals calculated using the Wilson method.

Interestingly, most of the analyzed pathogens showed significantly higher positivity rates in rats compared to mice. **Figure 3** presents the species-specific positivity rate aggregated over 2006–2025 for pathogens showing a statistically significant difference between mice and rats (p < 0.05), expressed as the proportion of positive samples with 95% confidence intervals calculated using the Wilson method. In particular, as shown, high positivity frequencies were observed in rats for *Syphacia muris* and *Mycoplasma spp*, with values markedly higher than those detected in mice. Conversely, *Clostridium piliforme, Chilomastix spp*, and *Trichomonas muris*, showed similar prevalence in rats and mice. *Trichomonas muris* represented a frequently detected agent in both species. For several bacteria, *Mycoplasma spp* and *Theiler’s encephalomyelitis virus*, positivity in mice was minimal or close to zero compared to the values observed in rats. Taken together, these findings demonstrate a differential distribution of the main infectious agents between the two species, confirming the presence of distinct health profiles in mouse and rat colonies.

**Figure 4:**
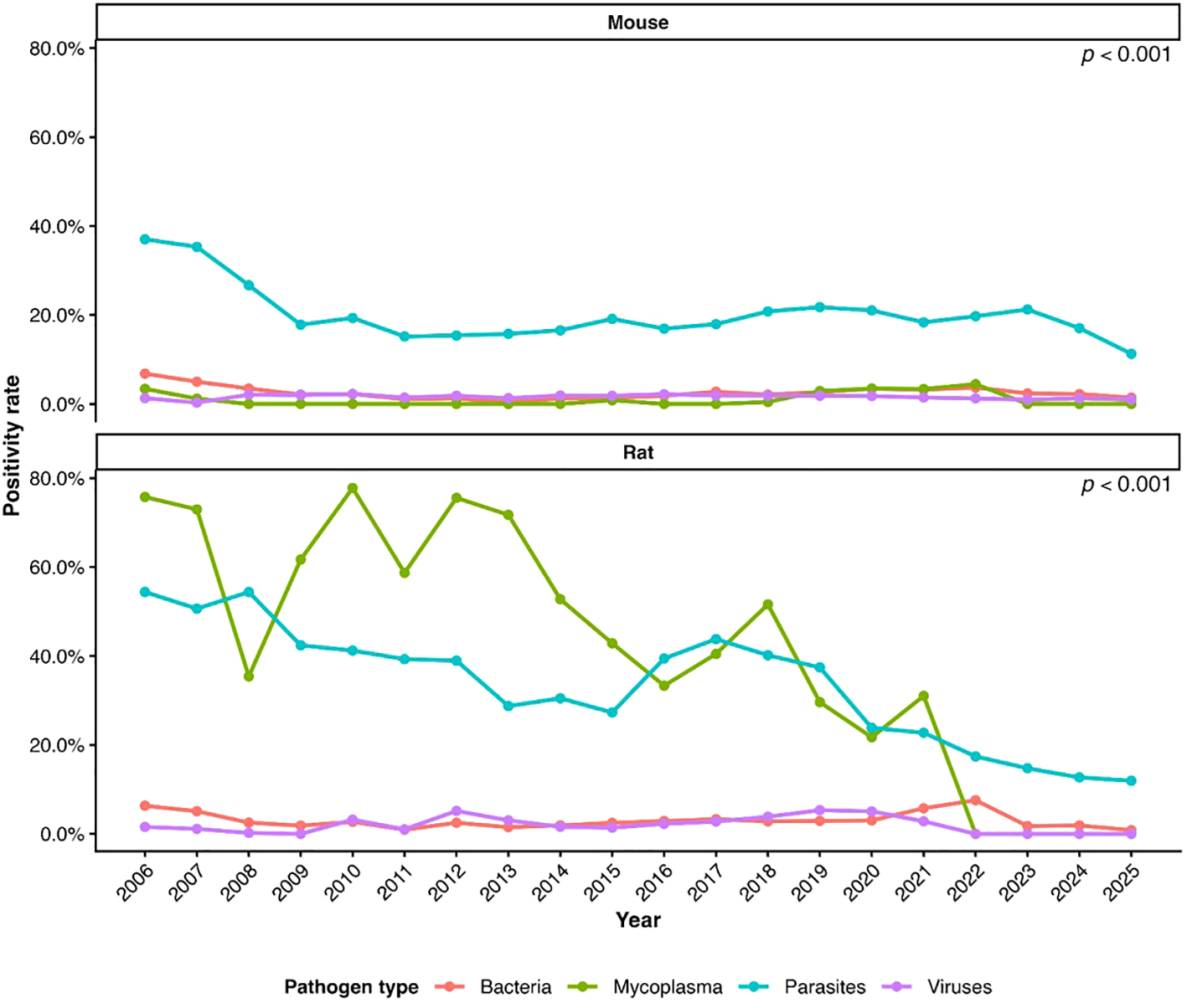
Contribution of different types of pathogens to yearly positivity rates. The graph summarizes the contribution of different pathogen categories (bacteria, mycoplasma, parasites, viruses) to yearly positivity rates in mice (top) and rats (bottom).

**Figure 4** shows the temporal trend of positivity rates stratified by pathogen category (bacteria, mycoplasmas, parasites, and viruses) in the two species studied over the entire observation period. In mice, parasites consistently represented the main cause of positivity throughout the study period. After high initial values in 2006–2007 (~35–37%), a marked reduction was observed until 2009–2011 (~15–20%), followed by a relatively stable phase with minor fluctuations and a moderate increase between 2017 and 2022. In the most recent years, a further decrease was recorded, reaching values close to 10–12% in 2025. Bacterial, viral, and mycoplasma-related positivities remained overall low throughout the study period (<5%), showing only modest temporal fluctuations without major variations. In rats, the pattern was more variable. Mycoplasma represented the predominant category in the early years of the study, with very high percentages (≈70–80%) and marked fluctuations until 2013–2014. Subsequently, a progressive decline was observed, leading to an almost complete disappearance of positivity after 2021. Parasites showed a decreasing trend over time, from values exceeding 50% in the early years to approximately 10–15% in the final period. Bacterial and viral infections generally remained at low frequencies (<10%), with transient increases, particularly for bacterial positivity around 2021–2022. Overall, the results highlight a different distribution of pathogens between the two species and a general reduction over time in the main causes of positivity, particularly evident in rats. As previously shown, parasites, especially endoparasites, were the microbial agents most frequently responsible for positive findings, in particular *Entamoeba muris, Giardia muris, Trichomonas muris*, and *Syphacia muris*.

**Figure 5:**
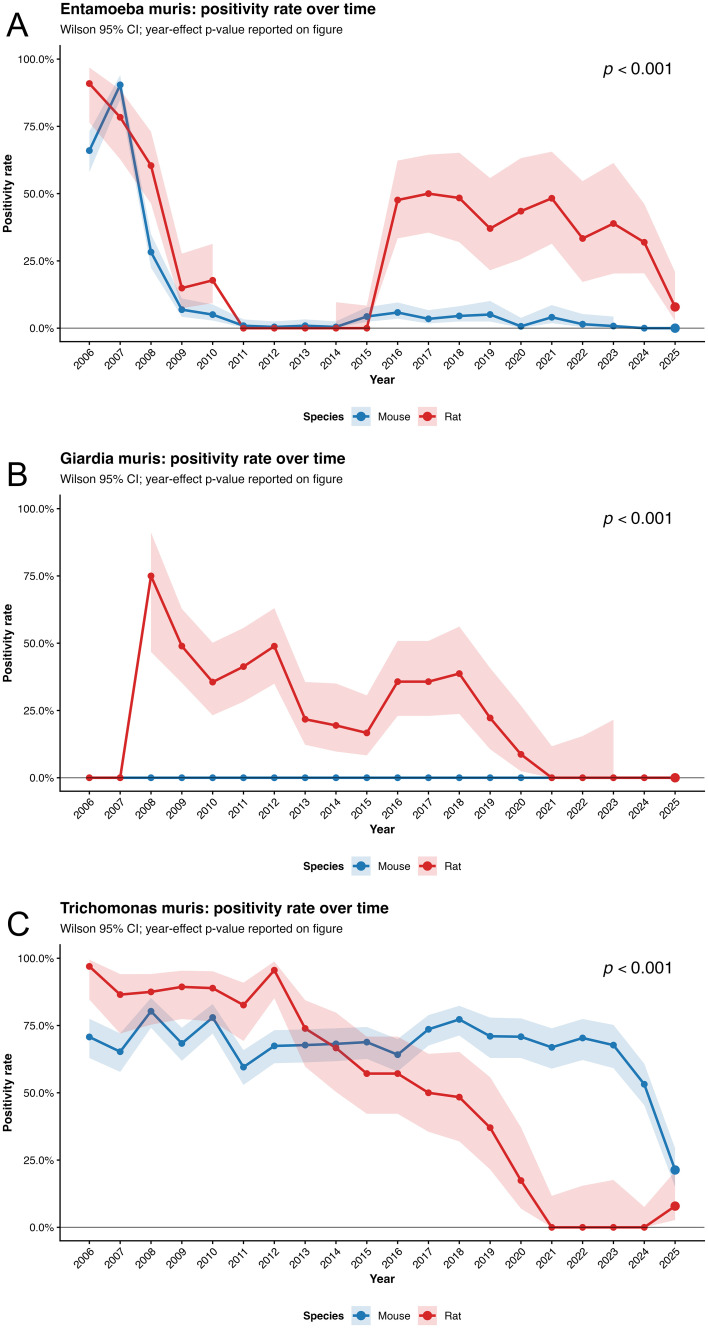
Temporal trend of positivity rates for endoparasites. **(A)** *Entamoeba muris,* **(B)** *Giardia muris,* **(C)** *Trichomonas muris*.

Therefore, temporal variations in the occurrence of these parasites were further investigated. **Figure 5** shows the temporal trends in positivity rates for four endoparasites, stratified by species, showing a significant effect of time (p < 0.001). As shown in **Figure 5A**, high positivity for *Entamoeba muris* was observed in the early years, followed by a marked decline to values close to zero in mice starting from 2010–2011. In rats, however, positivity remained variable around intermediate levels, with a decrease in recent years. For *Giardia muris* (**Figure 5B**), positivity was initially sporadic in mice and higher in rats, with a peak in the early years followed by a progressive decline until reaching zero in both species in the final observation period. *Trichomonas muris* (**Figure 5C**) showed persistently high positivity rates in mice for most of the study period, with a significant reduction only in the most recent years. In rats, a more pronounced and earlier decreasing trend was observed, with values declining to very low levels in the final period. Overall, the data demonstrate significant temporal variation for all analyzed parasites, with a general downward trend in positivity—particularly evident for *Giardia muris* and *Entamoeba muris*, as well as species-specific differences in infection dynamics.

**Figure 6:**
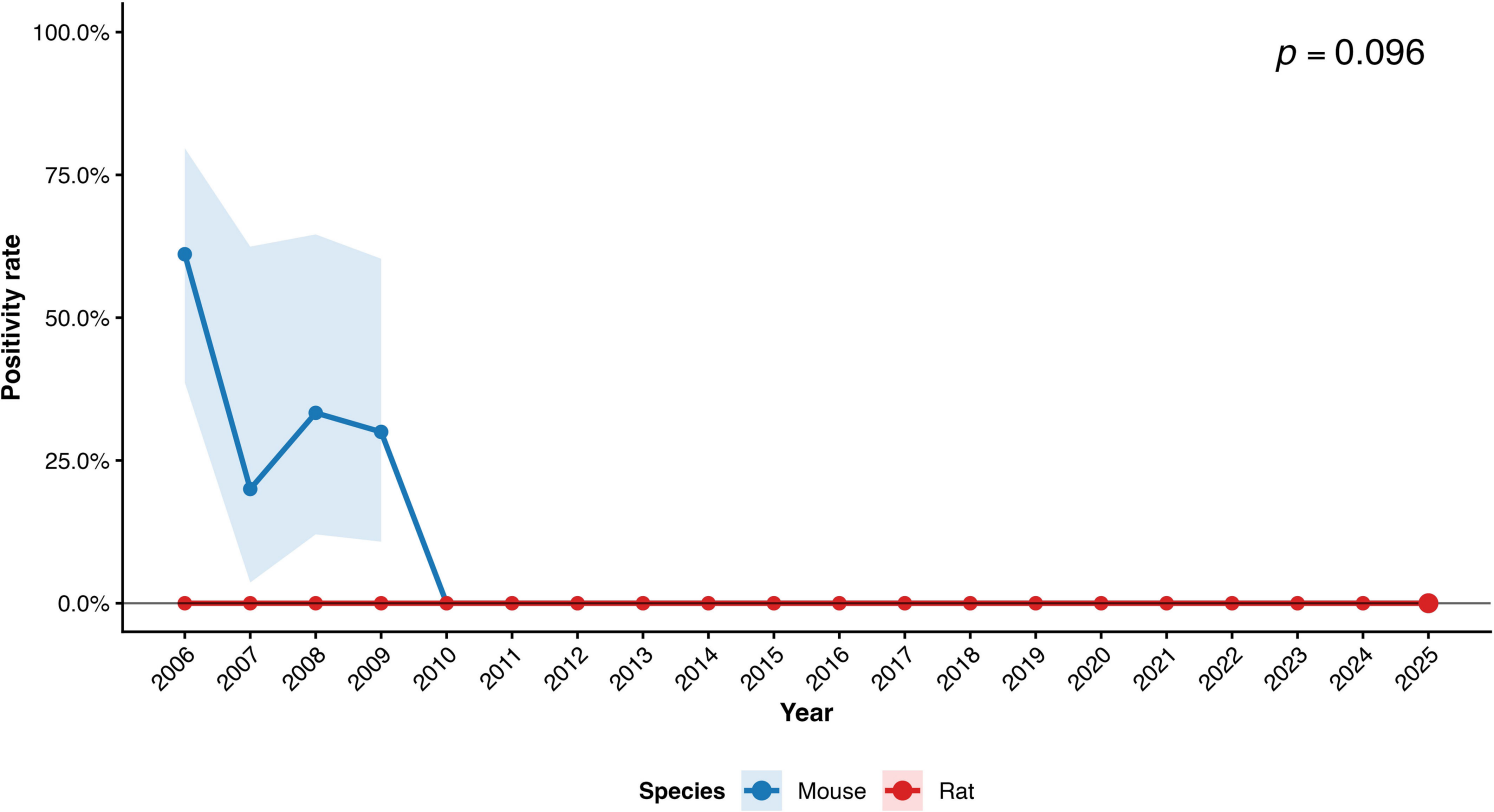
Temporal trend of positivity rates for ectoparasites. The graph presents yearly positivity rate over time for to *Myobia musculinus*, which was the only ectoparasite detected in early years in the animal facility and was eradicated.

A separate consideration concerns ectoparasites. **Figure 6** shows the temporal trend in positivity for *Myobia musculinus* in the monitored rodent colony. In mice, positivity was high in 2006 (approximately 60%) and declined over the following three years (≈20% in 2007; ≈30–35% in 2008–2009), reaching zero in 2010 following pharmacological treatments with ivermectin or permethrin administered on a rotational basis to small groups. In rats, positivity remained zero throughout the entire observation period. Analysis of the effect of year indicated a decreasing trend over time that did not reach statistical significance (p = 0.096; 95% Wilson CI).

**Figure 7:**
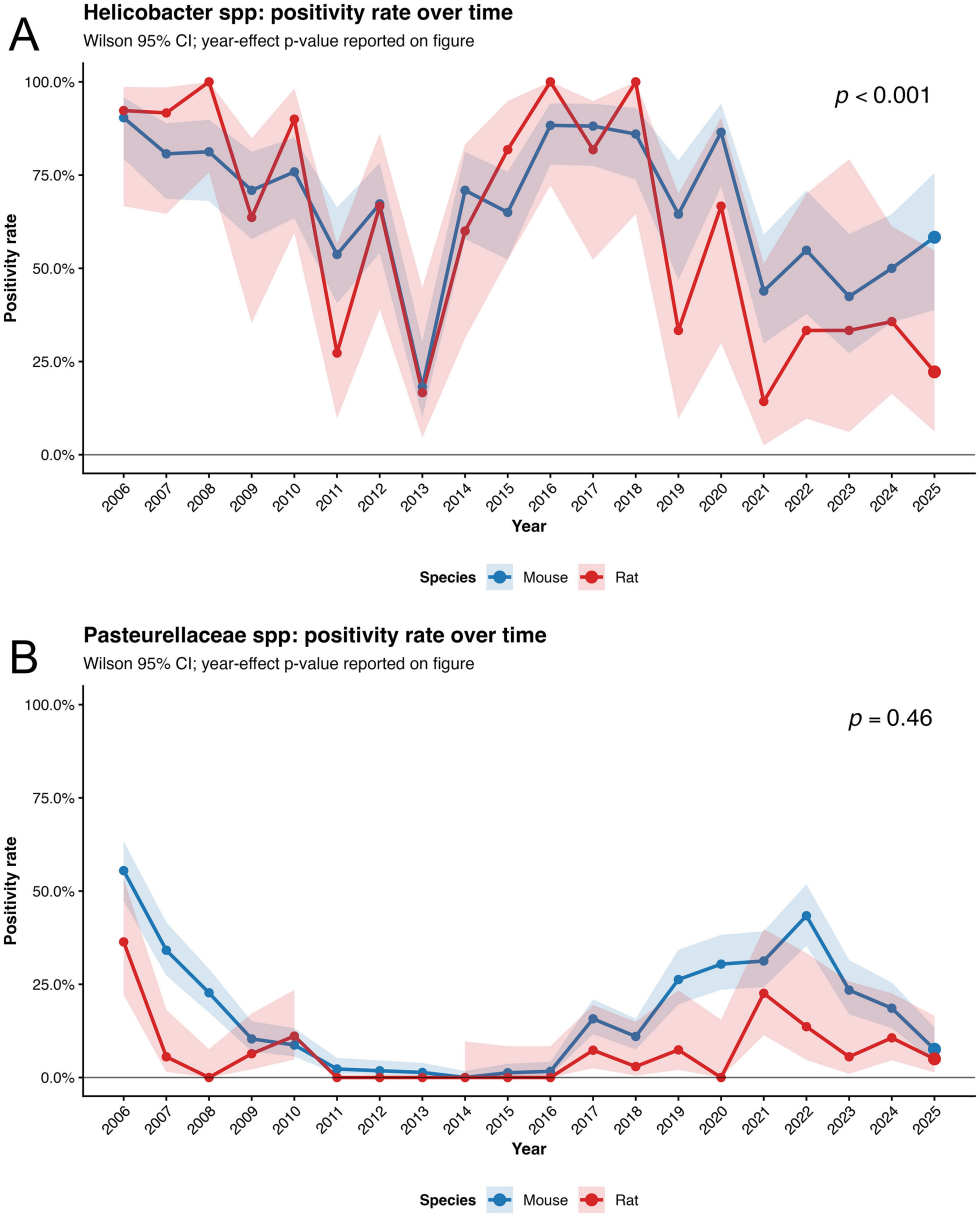
Temporal trend of positivity rates for bacteria. **(A)** *Helicobacter spp*, **(B)** *Pasteurellaceae* spp.

**Figure 7A** shows high positivity rates for *Helicobacter spp* in both species during the first part of the observation period, frequently exceeding 70–80%, with wide interannual fluctuations. After a marked decline around 2013, positivity increased again, reaching new peaks in subsequent years, followed by a more evident reduction in the most recent period. The analysis demonstrated a significant effect of time (p < 0.001). Overall, rates were comparable between the two species. For *Pasteurellaceae spp* (**Figure 7B**), an initial decline in positivity was observed in both species, reaching values close to zero in the early 2010s. In subsequent years, re-emergence occurred, generally with low values, more evident in mice than in rats, followed by a further decrease in the final period. Unlike what was observed for *Helicobacter spp*, the effect of time was not statistically significant (p = 0.46). Overall, **Figure 7** highlights variable temporal trends for both agents, with a more pronounced and statistically significant dynamic for *Helicobacter spp*, and more evident quantitative differences between species for *Pasteurellaceae* spp.

**Figure 8:**
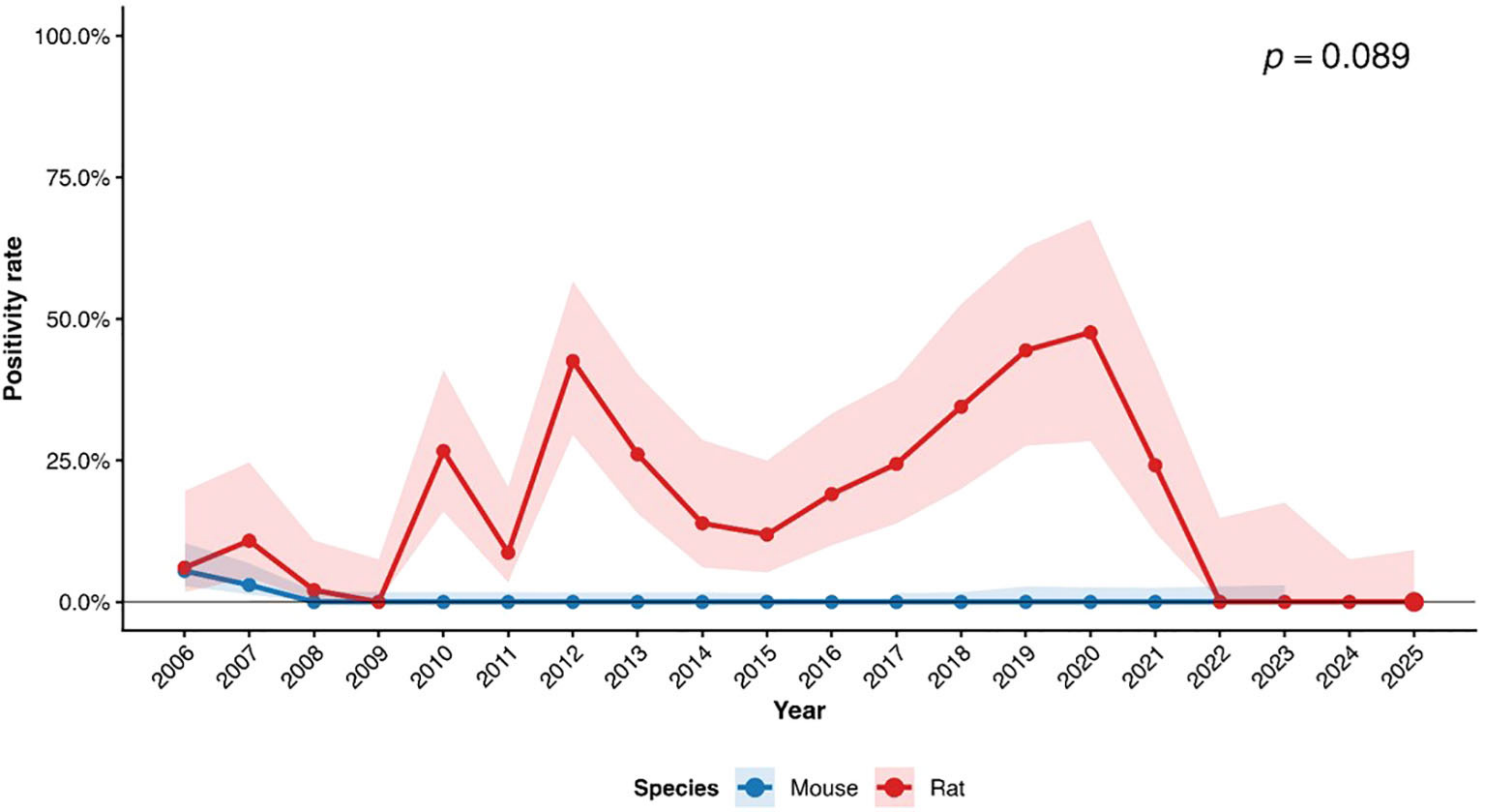
Temporal trend of positivity rates for *Theiler’s encephalomyelitis virus. GDVII virus* was absent in mice and was eradicated in rats in recent years.

**Figure 8** shows the temporal trend in positivity rates for *Theiler’s encephalomyelitis virus*. In mice, a low positivity was limited to the early years of observation and absent thereafter. In rats, intermittent positivity was observed starting in 2006, with an increase between 2011 and 2021 and a peak around 2019–2021, followed by a return to zero values in the most recent years. The effect of year indicated a temporal trend that did not reach statistical significance (p = 0.089; 95% Wilson CI).

**Figure 9:**
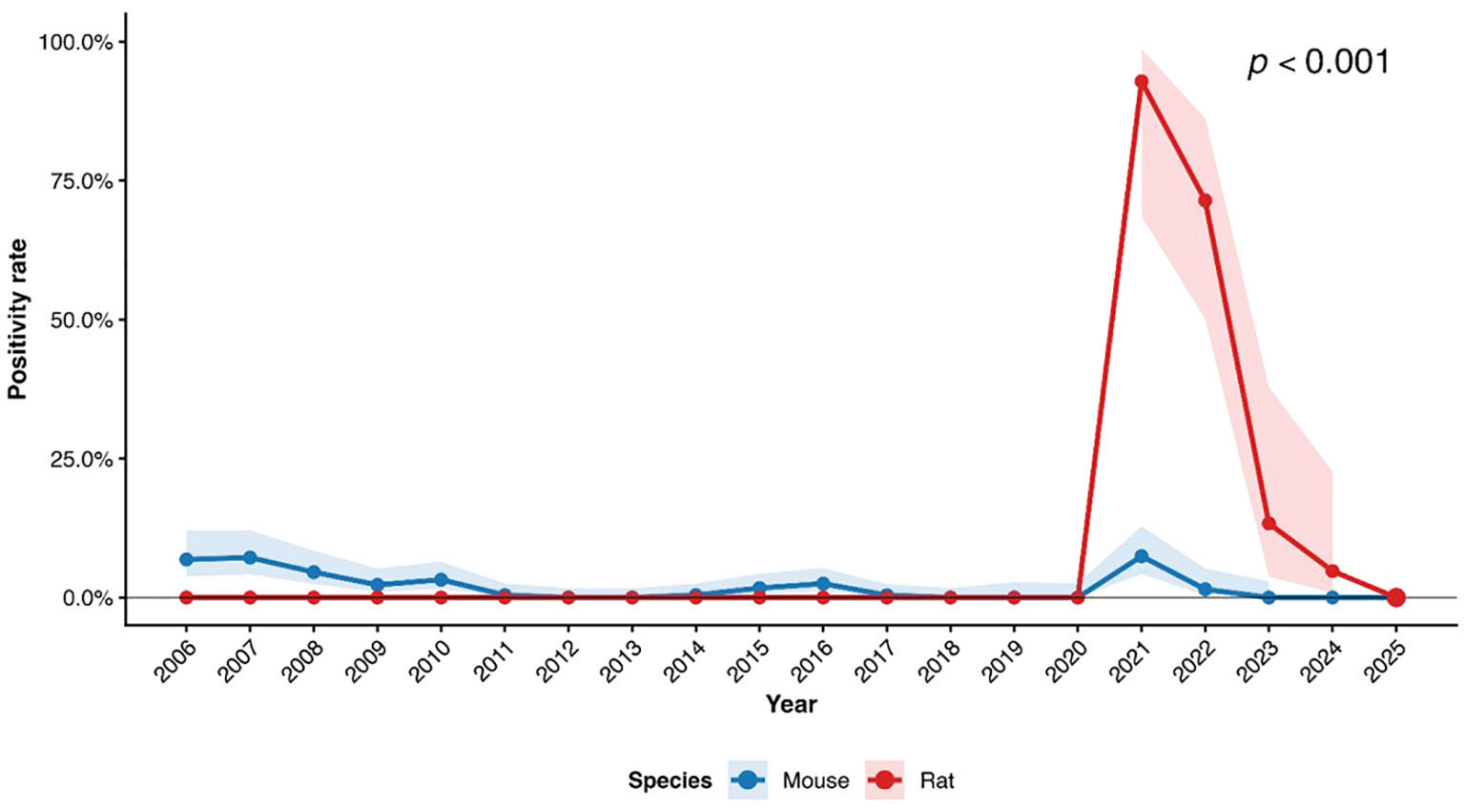
Yearly positivity rates for *Proteus* spp. The graph shows an occasional infection by this gram-negative bacillus and its rapid containment following pharmacological treatments administered on a rotational basis to small groups of animals.

**Figure 9** illustrates the temporal trend in positivity for *Proteus spp* in the monitored colony between 2006 and 2025. In mice, positivity was generally very low and sporadic in the early years (2006–2010), with values below 10%, and subsequently declined to nearly zero, except for a slight increase in 2021, followed by a return to null values. In rats, positivity remained absent until 2020. In 2021, a marked increase was observed, with a peak approaching 90%, followed by a progressive decline in 2022–2024, reaching zero again in 2025. Analysis of the effect of year demonstrated a statistically significant temporal variation (p < 0.001; 95% Wilson CI), indicating a substantial change in prevalence over time, particularly in the rat population in the most recent years.

**Figure 10:**
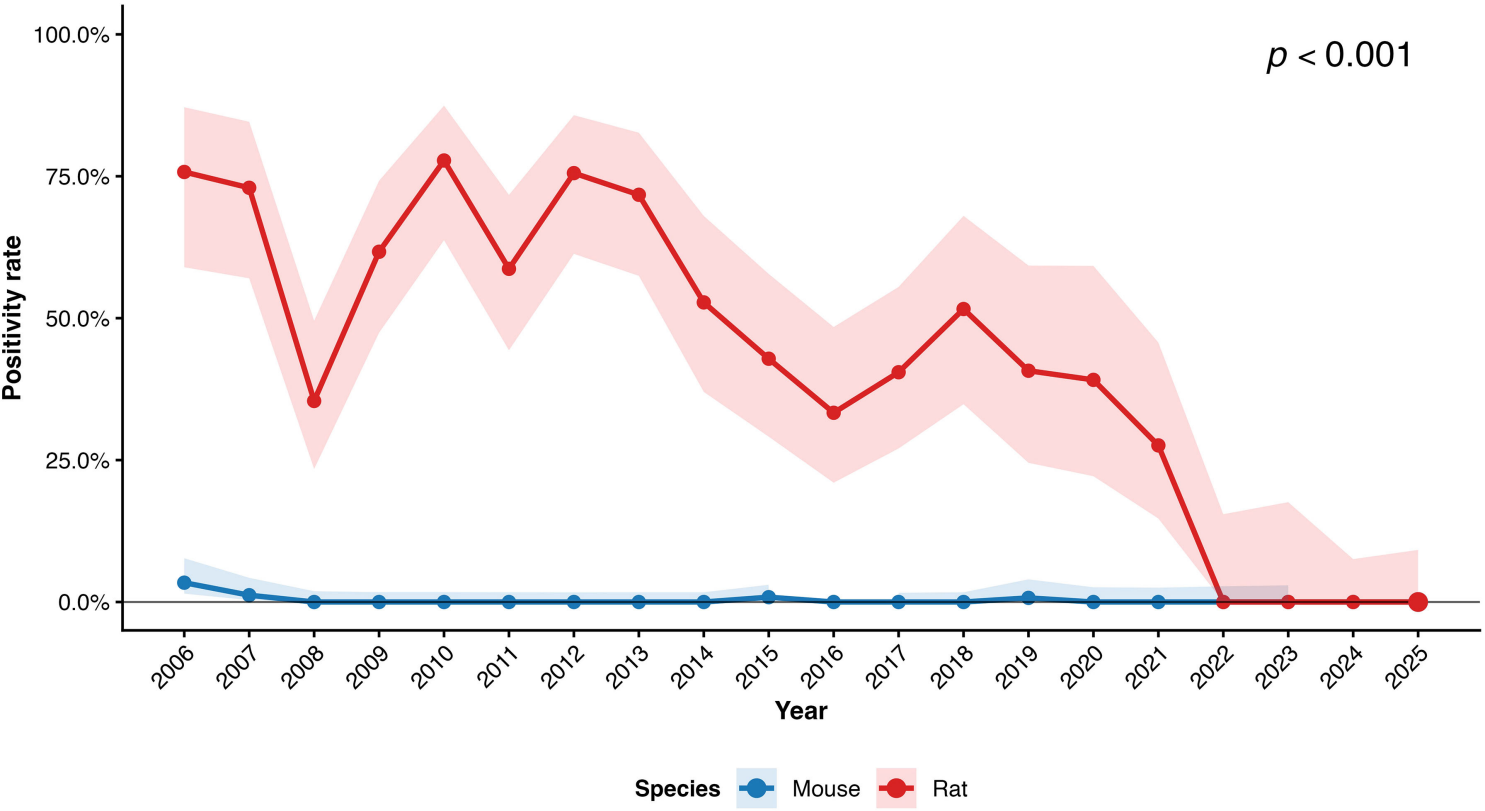
Yearly positivity rates for *Mycoplasma* spp. The graph presents the eradication of this pathogen, obtained by means of management interventions (isolation of positive animal groups).

Longitudinal health monitoring also revealed a marked difference in the prevalence of *Mycoplasma spp* between the two housed rodent species (**Figure 10**). Rats showed historically high positivity rates, with substantial fluctuations between 2006 and 2013 and peaks exceeding 75%. Starting in 2012, a progressive and statistically significant downward trend was observed (p < 0.001), leading to complete negativization of the rat colony from 2022 onward, a status maintained until the end of the observation period. In contrast, in mice, *Mycoplasma spp* prevalence remained consistently close to 0% throughout the entire 20-year period.

**Figure 11:**
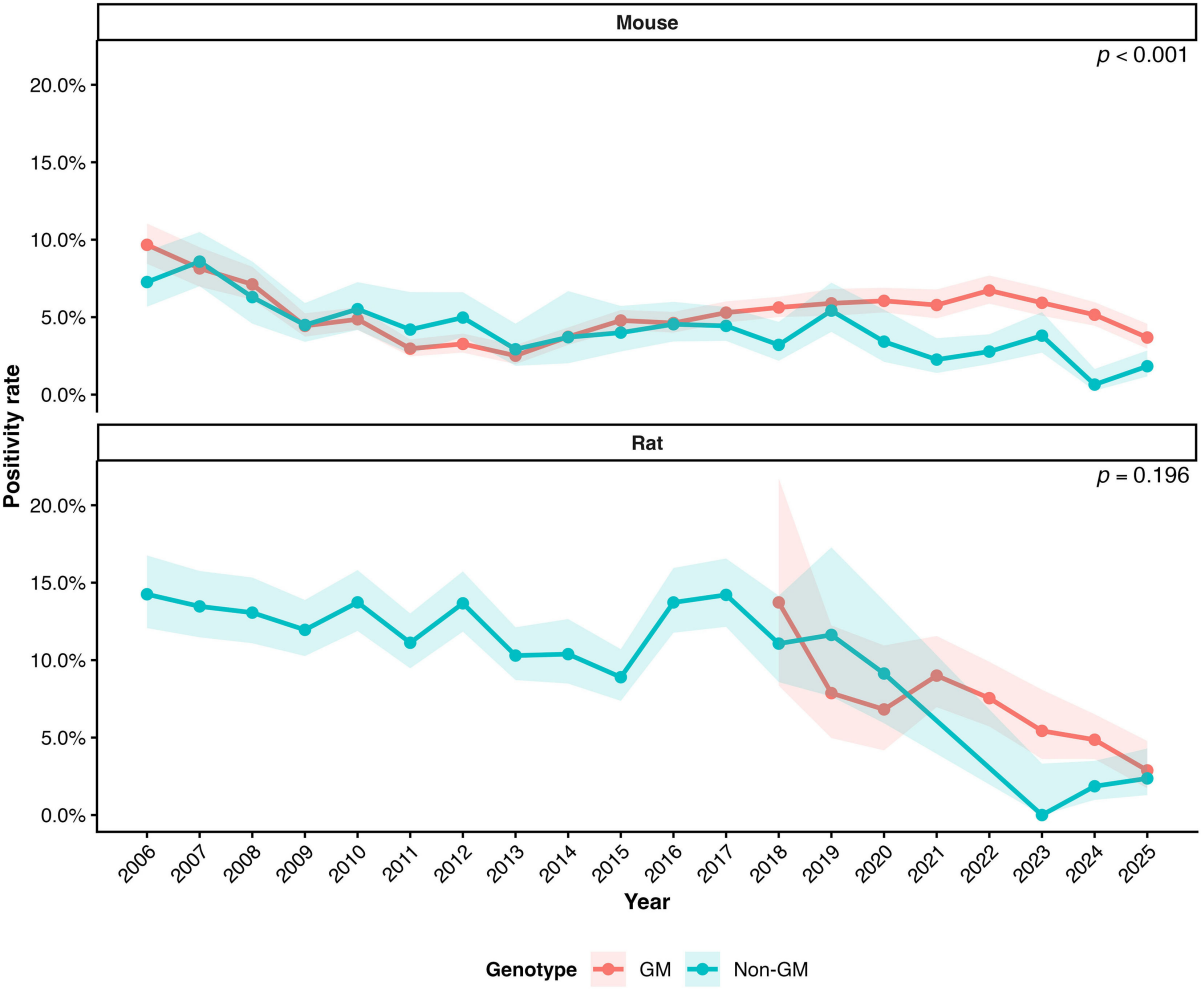
Temporal trend of positivity rates in GM *vs* non−GM animals. Yearly positivity rates stratified by species (top, mouse; bottom, rat), with model−based p−values adjusted for year.

**Figure 11** shows the temporal trend in overall pathogen positivity rates stratified by genotype (GM *vs* non-GM) in mice and rats over the 2006–2025 period, with genotype-effect p-values adjusted for year reported separately for each species. In mice, positivity rates were consistently comparable between GM and non-GM strains in the early years, followed by a gradual divergence from approximately 2014 onward, with GM animals generally showing slightly higher positivity rates than non-GM animals. While both groups exhibited a declining trend over time, the reduction was more pronounced in non-GM mice in the most recent years, reaching values close to 1–2% by 2024–2025. The genotype effect was statistically significant in mice (p < 0.001), indicating a consistent difference in positivity rates between GM and non-GM animals after adjustment for time. In rats, positivity rates were initially high in non-GM animals (approximately 12–15%) and showed marked fluctuations until 2017. From 2019 onward, a progressive decline was observed, with values approaching zero in the final years of observation. The genotype effect was not evaluable for rats, since GM strains were introduced only in 2018. Their positivity rates showed a steady decline over time. Overall, **Figure 11** indicates a progressive reduction in pathogen positivity in both species, with a significant genotype-associated difference observed only in mice.

**Figure 12:**
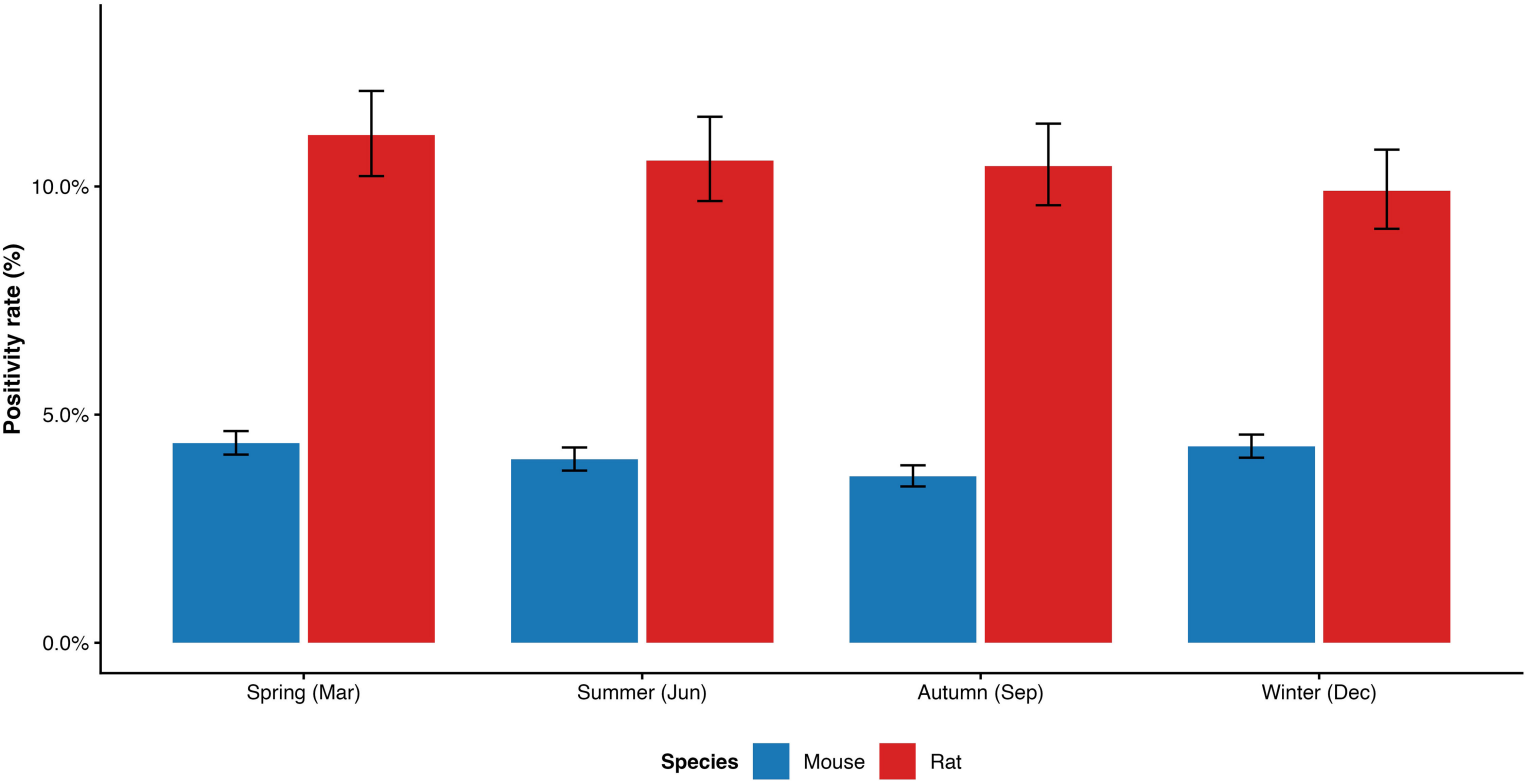
Seasonality analysis of positivity rates in mice and rats. The seasonality analysis is restricted to routine surveillance months (March/June/September/December). The graph presents significant effect of seasonality in mice, but not in rats.

The analysis of the seasonal distribution of positivity rates revealed divergent patterns between the two monitored species (**Figure 12**). In mice, a statistically significant seasonal variation was observed (p < 0.001). The highest positivity rates were recorded in spring (March) and winter (December), with values close to 4.5%. A slight but consistent decline was detected during the summer and autumn months, with a minimum of 3.7% in September. In contrast, rats did not show a significant seasonal pattern (p = 0.302). Although absolute positivity rates were markedly higher than those observed in mice (ranging between 10% and 11.2%), prevalence remained substantially stable throughout the year, with no statistically significant peaks across seasons. These data indicate a stable annual distribution in rats and a significant seasonal variation in mice.

## 4 Discussion

The comparison between the overall positivity rate trend (**Figure 1**) and the analysis by pathogen type (**Figure 2**) indicates that the temporal variations observed in the two species during the study period are mainly attributable to changes in the prevalence of the dominant pathogens. In rats, the high positivity levels recorded in the initial phase of the study are consistent with the high frequency of mycoplasma infections and, secondarily, parasitic infections. The progressive reduction in the overall positivity rate over time coincides with the marked decline in mycoplasma positivity, suggesting an improvement in the effectiveness of health monitoring and microbiological control strategies implemented over the years (Bracken et al., 2017; Carriquiriborde et al., 2020).

**Figure 13:**
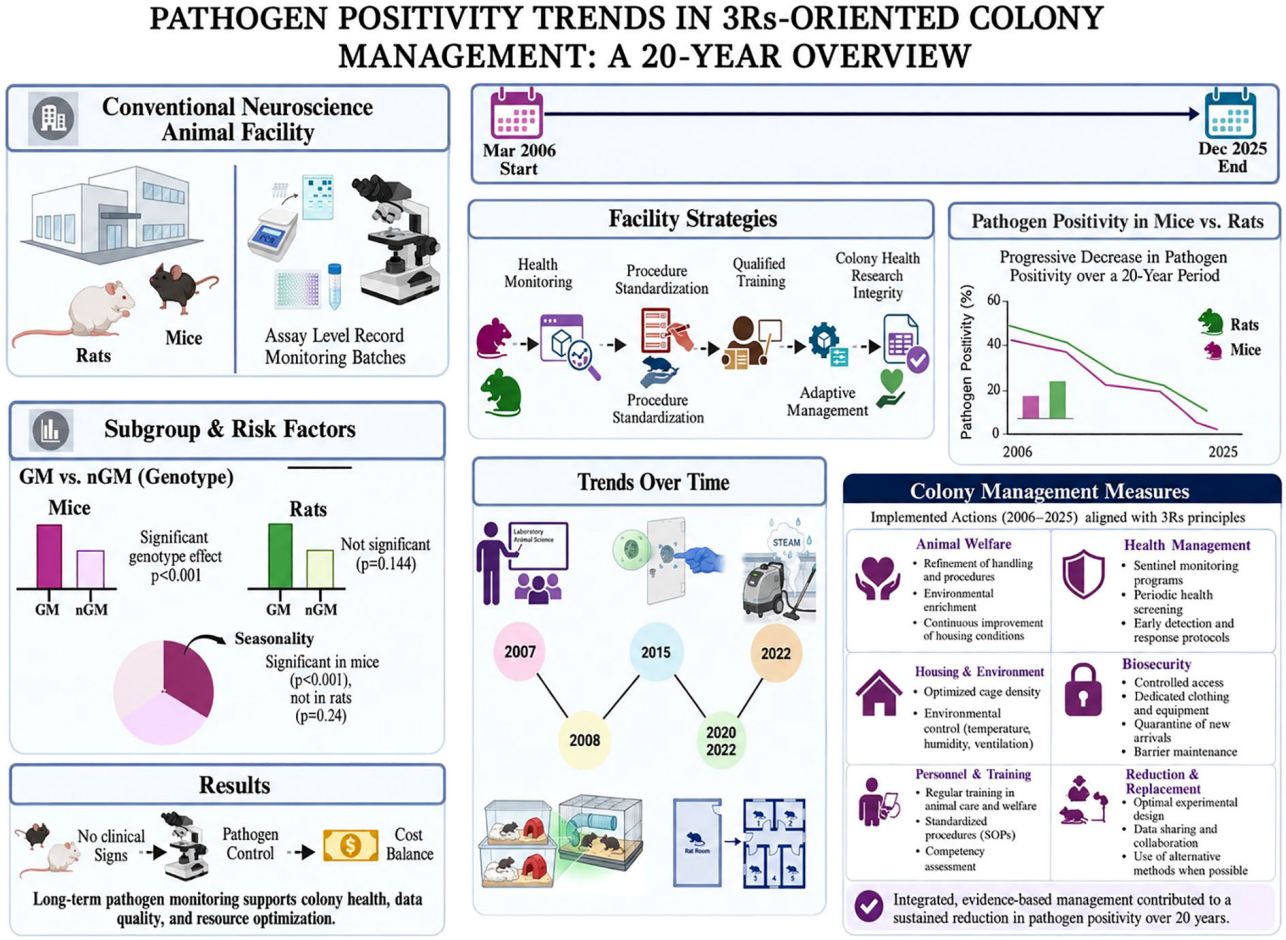
Graphical overview of the 20-year sentinel-based health-monitoring programme in a conventional neuroscience animal facility. The schematic summarizes the health-monitoring programme implemented in a centralized conventional neuroscience animal facility housing mice and rats, and illustrates the construction of the retrospective dataset. The facility strategies panel summarizes the main contextual and management components considered during the study period, including continuous health surveillance, qualified staff training, adaptive management procedures, and actions aimed at supporting colony health and research-data interpretation. The annual pathogen positivity panel shows the longitudinal output generated by the surveillance system in mice and rats. Positivity was defined as positives/(positives + negatives) and is displayed as yearly aggregated data with 95% Wilson confidence intervals. Temporal trends were evaluated using binomial logistic regression on yearly counts, with pathogen-level *p*-values controlled by the Benjamini–Hochberg false discovery rate. Subgroup and risk-factor analyses included species, genotype status, and seasonality. The timeline summarizes key management and facility changes introduced during the study period: structured staff training (2007), environmental-enrichment expansion (2008), fingerprint-based access control to improve traceability and restrict entry (2015), increased physical separation of rat colonies through room expansion (2020–2022), and periodic environmental steam sanitization (2022). These elements were considered as contextual factors for interpreting longitudinal pathogen-positivity patterns. The Colony Management Measures panel should be interpreted as a conceptual framework rather than as a measured outcome of the present study. In this context, health monitoring is presented as an operational component of colony health management that may be guided by 3Rs and Culture of Care principles when translated into risk-based decisions, veterinary oversight, staff communication, targeted management actions, and continuous improvement. The conclusions panel summarizes the main interpretative message of the study: structured health-monitoring records can support animal health management, biosafety, and research-data interpretation in a conventional facility, while animal welfare, Culture of Care, scientific quality, and causal effects of specific interventions were not directly measured. Created with BioRender.com.

In this regard, **Figure 13** provides a visual summary of the approach adopted in the considered conventional facility, illustrating the evolution of health monitoring strategies, their integration with the principles of the 3Rs and the Culture of Care, and the positive impact observed in the sustained improvement of the health status of the colonies. The figure also highlights how management effectiveness is closely linked to scientific quality and animal welfare, confirming the interdisciplinary and multi-level nature of the ethical and scientific management applied over time (Amarasekara et al., 2022; Calvillo, 2022).

A particularly relevant aspect of described management strategy was the use of targeted and selective interventions on specific groups of animals, whenever treatment of the entire colony was not feasible due to scientific, organizational, or economic reasons. The separation and targeted monitoring of subgroups allowed for the eradication of critical pathogens such as *Myobia, Mycoplasma*, and the *Theiler’s encephalomyelitis virus*, significantly reducing their spread without resorting to extensive treatments that could interfere with experimental protocols or increase animal use. This strategy, replicable in other conventional facilities facing similar scientific and/or organizational and economic challenges, represents an effective model to balance biosafety, sustainability, and scientific continuity (Buxbaum et al., 2011; Peres and Roe, 2022).

In mice, the overall positivity rate shows smaller variations, reflecting a relatively stable pathogen distribution over time. In this species, positivity is mainly associated with persistent parasitic infections, while other pathogen categories maintain a low incidence throughout the observed period. The moderate fluctuations in the overall trend thus seem to depend primarily on quantitative variations in the parasitic component rather than substantial changes in the health profile (Buchheister and Bleich, 2021).

Taken together, these results highlight species-specific epidemiological dynamics, with a marked transition toward a global reduction in positivity in rats compared to a more stable trend in mice. Such differences could reflect variations in host susceptibility, colony management practices, or the differential impact of biosafety measures (Drake et al., 2008). These data emphasize the importance of continuous and species-adapted health surveillance programs to ensure controlled microbiological conditions and improve the reproducibility of physiological studies based on animal models (Hampshire and Gilbert, 2019; Rinwa et al., 2024).

The analysis of species-specific differences in the positivity of individual pathogens (**Figure 3**) provides an additional interpretative level to the temporal trends observed in the previous graphs. In particular, the higher overall positivity found in rats throughout much of the study period appears consistent with the higher prevalence of numerous infectious agents in this species, including *Mycoplasma spp*, intestinal parasites, such as *Syphacia muris* and *Trichomonas muris* (Bracken et al., 2017; Carriquiriborde et al., 2020).

This distribution suggests that the reduction in the overall positivity rate in rats observed in recent years may primarily reflect the progressive control of pathogens historically more widespread in this species, rather than a uniform decrease in infectious risk (Mähler et al., 2014). Conversely, in mice, the generally lower positivity levels for most analyzed agents align with the more stable temporal trend observed in the overall rate, indicating a generally more homogeneous health profile over time.

Together, these findings support the hypothesis that differences in epidemiological trends between mice and rats are driven by specific host–pathogen associations. These differences are particularly relevant in the context of physiological research, as the species-dependent presence of subclinical infectious agents can contribute to biological variability and influence experimental reproducibility (Mähler et al., 2014; Prager et al., 2019; Compton, 2020). The integration of temporal analysis and species-specific pathogen evaluation thus underscores the importance of species-targeted health surveillance strategies (Mähler et al., 2014).

The longitudinal analysis (**Figure 5**) shows a clear improvement in health status over the considered period, with a progressive reduction in positivity for most monitored endoparasites. This result is particularly relevant given that no broad-spectrum anthelmintic or antiparasitic treatments were performed on all colonies to avoid interference with experimental protocols (Clifford and Watson, 2008). Improvement was associated with optimization of housing conditions, strengthening of access and use procedures in the animal facility (barriers, flows, personal protective equipment, sanitation), and isolation/containment of positive colonies, confirming the effectiveness of biosafety and management measures (Shek, 2008; Mähler et al., 2014).

Differences observed between mice and rats suggest different sensitivity and susceptibility to the considered agents (Baker, 1998; Barthold et al., 2016). In particular, rats showed higher and more persistent positivity rates for some parasites (e.g. *Giardia muris* and *Entamoeba muris*) compared to mice, indicating a possible greater susceptibility or a greater capacity to maintain infection circulation within colonies. Conversely, in mice, a more rapid or marked reduction in positivity was observed in several cases, although for *Trichomonas muris* the decrease occurred later. These differences may reflect species-specific variables related to behavior, housing density, group dynamics, or immune response (Barthold et al., 2016; Compton, 2020).

It is known that eradicating oro-fecally transmitted endoparasites in conventional colonies is a management challenge, especially in the absence of systematic treatments, due to environmental persistence of infective forms and the possibility of reinfection (Baker, 1998; Pritchett-Corning et al., 2009). In this context, the observed progressive reduction demonstrates the importance of an integrated and sustained approach over time.

It should also be noted that the monitored colonies consisted of immunocompetent rodents, which, despite occasionally high positivity levels, never showed clinical signs attributable to the detected parasitic agents. This confirms that such infections can proceed sub-clinically and reinforces the need for continuous monitoring even in the absence of obvious symptoms (Baker, 1998; Shek, 2008).

The observed trend for *Helicobacter spp* (**Figure 6**) shows that this agent represents a persistent critical issue within the monitored colonies. The high positivity percentages recorded for several years, along with wide interannual fluctuations, suggest stable and difficult-to-eradicate circulation, likely favored by oro-fecal transmission and possible chronic colonization of immunocompetent animals (Whary and Fox, 2006). The subsequent reduction in rates in the final phase of the observation period indicates progressive improvement, plausibly related to the strengthening of management and biosafety measures rather than systematic therapeutic interventions. This data confirms that controlling *Helicobacter spp* in conventional colonies requires consistent structural and organizational interventions over time rather than isolated actions (Pritchett-Corning et al., 2009).

Regarding *Pasteurellaceae spp*, the absence of a statistically significant temporal trend, despite contained and generally low fluctuations, suggests a sporadic or intermittent presence, probably linked to isolated episodes rather than stable endemic spread (Barthold et al., 2016). The greater variability observed in mice compared to rats may reflect species-specific differences in susceptibility, group dynamics, or housing conditions.

Overall, the data indicate that even in the absence of broad-spectrum treatments, which on some occasions cannot be implemented to preserve the integrity of experimental protocols, improvement of environmental conditions, access control, strict adoption of barrier procedures, and isolation of positive colonies can lead to a substantial reduction in bacterial agent circulation (Pritchett-Corning et al., 2009; Mähler et al., 2014). However, the difficulty of achieving complete eradication remains evident, especially for microorganisms capable of establishing subclinical chronic infections in immunocompetent animals, which never exhibited clinical signs attributable to these pathogens but may serve as infection reservoirs and potential sources of experimental variability (Baker, 1998; Prager et al., 2019).

### 4.1 Continuous health monitoring and animal health management

In a conventional facility, regular and continuous health monitoring should be interpreted primarily as a microbiological surveillance and colony health-management tool. By allowing the identification of longitudinal variations, clustered patterns of microbial circulation and potential health risks, systematic surveillance can support timely and proportionate management decisions (Besselsen et al., 2008; Pritchett-Corning et al., 2009; Palacios et al., 2010). In this sense, HM may contribute to Refinement mainly through the protection of animal health and the early detection of health-related risks (Russell and Burch, 1959; Mähler et al., 2014; Calvillo, 2022; Eichner and Smith, 2023). In addition, HM may also contribute to Reduction by limiting the risk of undetected infections that could compromise experimental outcomes, require repetition of studies, or lead to unnecessary loss and replacement of animals or valuable colonies. From a scientific perspective, a documented and stable microbiological status supports data interpretability, reproducibility, and the overall quality of *in vivo* research.

In the present retrospective study, routine clinical and behavioural observations of sentinel animals are interpreted as contextual welfare-related information, not as a comprehensive assessment of animal welfare or affective state (Müller et al., 2022; Merley et al., 2022). In fact, experimental studies in mice suggest that dirty or soiled bedding exposure may have context-dependent effects on sentinel animals, ranging from reduced body weight gain to reduced abnormal repetitive behaviours, depending on study design, exposure duration, bedding composition, strain, sex, and parameters assessed (Müller et al., 2022; Merley et al., 2022).

### 4.2 Microbiological status, biological variability and research data interpretation

Many effects depend on context: strain, genetic background, age, environmental load, and variable interactions (Peres and Roe, 2022). In this scenario, continuous monitoring plays a crucial role in transparency and contextualization. Knowing microbiological status allows interpretation of experimental data within a defined framework, reducing the risk of unrecognized bias and strengthening reproducibility (Prager et al., 2019; Voelkl et al., 2020). In a conventional facility, documenting the microbiological status of co-housed experimental groups can help contextualize experimental findings and identify potential biological confounders, without implying that absolute microbiological sterility is required.

### 4.3 Pathogen-specific management and proportionate corrective actions

Management of microbiological positivity in conventional colonies requires pathogen-specific risk assessment and proportionate corrective actions. In the described context, all animals were immunocompetent and clinically asymptomatic. Depending on the agent involved, the clinical status of the animals, the type of research being conducted and facility policy, possible responses may include intensified monitoring, isolation or containment of positive groups, rederivation, targeted treatment where appropriate, or other management measures (Pritchett-Corning et al., 2009). Large-scale elimination should be avoided, since it would entail euthanasia of a high number of healthy animals, with subsequent need for recolonization and overall increase in animal use (Festing and Wilkinson, 2007). Such a strategy would be hardly compatible with the 3Rs principles.

When pharmacological treatment is considered, its potential benefits should be weighed against possible effects on microbiota composition, immune status, behaviour, and experimental endpoints (Bleich and Hansen, 2012; Ericsson et al., 2018). Broad-spectrum treatments may introduce additional biological variables and should therefore be evaluated in relation to the pathogen involved, the clinical status of the animals, the experimental models in use, and the feasibility of alternative management measures. In neuroscience models focused on anxiety, cognition, or seizure susceptibility, even subtle alterations may represent a greater confounding factor than controlled circulation of agents with low clinical pathogenicity. In this perspective, management decisions in conventional colonies should not be framed simply as a choice between treatment and non-treatment, but rather as pathogen-specific, risk-based decisions that balance animal health, potential welfare implications, scientific interference, biosafety, and long-term sustainability (Pritchett-Corning et al., 2009; Vitale and Ricceri, 2022).

### 4.4 Health monitoring reports within welfare-oriented colony management

Health-monitoring reports should be interpreted primarily as operational records supporting colony health management. They document surveillance activities, diagnostic findings and management decisions, and can help facility staff, researchers and veterinarians identify health risks requiring timely action. Their value therefore lies not only in the technical recording of pathogen-monitoring data, but also in the way this information is reviewed, communicated and translated into practical management decisions. The 3Rs and Culture of Care principles may guide how health monitoring information is collected, interpreted and used within the facility to support responsible and welfare-oriented colony management. In this perspective, health reports may contribute to a structured decision-making process by promoting veterinary oversight, staff communication, timely corrective actions, training, shared responsibility and continuous improvement. When embedded within such a broader institutional framework, health monitoring records can support the control of relevant biological variables and provide contextual information that may contribute to animal health, personnel safety and the reliability of experimental conditions.

This occupational-health dimension is consistent with recent reviews on laboratory-associated zoonotic parasitic infections, which emphasize that biological risks may be underestimated or underreported and that prevention requires risk assessment, good laboratory practices, appropriate personal protective measures, standardized disinfection procedures, and updated risk indicators (Sini et al., 2023). Thus, health-monitoring information may also support personnel biosafety when integrated with staff training and documented procedures.

Over twenty years of the present study, the management approach has evolved into a preventive and multilevel strategy integrating daily clinical monitoring, periodic health checks in accordance with FELASA guidelines, structural and organizational improvements, continuous staff training, and promotion of a Culture of Care (Burkholder et al., 2012; Mähler et al., 2014; Montanari et al., 2024). The inclusion of genetically modified strains and animals already present in colonies may have increased the representativeness of the monitoring programme without requiring additionally dedicated animals, in line with Reduction. At the same time, targeted interventions on isolated groups allowed containment or significant reduction of specific agents without resorting to drastic measures (Pritchett-Corning et al., 2009; Eichner and Smith, 2023).

The controlled persistence of some microorganisms in immunocompetent and clinically asymptomatic colonies does not necessarily represent management failure but may reflect a proportionate strategy balancing biosafety, animal welfare, and scientific continuity (Baker, 1998; Buchheister and Bleich, 2021; Peres and Roe, 2022).

## 5 Conclusions and future directions

The reported longitudinal experience suggests that continuous health monitoring represents a dynamic instrument of animal colony management. It allows identification of temporal trends, implementation of targeted interventions, early identification of health-related issues, preservation of genetically valuable lines, and reduction of additional animal use due to experimental repetitions (Festing and Wilkinson, 2007). Ultimately, regular health surveillance is a key element integrating animal health and research data interpretation.

Due to its retrospective nature, the present study could not provide evidence of a causal relationship between progressive management interventions and the decrease in pathogen positivity observed over time. Despite this limitation, the data may suggest that sustained institutional commitment to animal health, personnel well-being, biosafety, transparent communication, and documented colony management can support a responsible and progressively refinement-oriented approach in a conventional facility.

In the future, the facility will work to implement environmental health monitoring strategies, such as sentinel-free soiled bedding testing and direct colony sampling, to replace the need for live animal sentinels and further refine the care of colony animals. It will therefore be useful to monitor changes in pathogen positivity in the coming years.

Specifically, in the current monitoring workflow, direct colony sampling with PCR-based analyses performed on faecal pellets collected from bedding material, together with oropharyngeal and skin swabs and blood sampling, has reduced the need for terminal use of sentinel animals, while maintaining reliable surveillance of the health status of colonies housed in the conventional facility. Transition to sentinel-free soiled bedding techniques is consistent with the need to further refine sentinel-based programmes by considering not only diagnostic performance but also potentially stressful repeated exposure to soiled-bedding, oral swabs and blood sampling procedures on sentinel animals (Müller et al., 2022; Merley et al., 2022). In the future, full transition to sentinel-free soiled bedding techniques is expected to replace the need for sentinel animals altogether, while preserving high standards of microbiological surveillance and colony health management.

Notably, the development of Home Cage Monitoring systems and technologies based on big data has opened new perspectives for advanced, continuous, and non-invasive surveillance (Fuochi et al., 2024). The 24-hour/7-day collection of behavioural and physiological parameters directly within the home cage may allow earlier detection of changes in health or welfare status and provide objective tools for complementary welfare assessment.

In this perspective, a calibrated and evidence-based health monitoring approach could be interpreted as an operational practice that can be guided by ethical, welfare-oriented principles to balance animal health, biosafety, scientific results, and operational sustainability (Russell and Burch, 1959; Marx et al., 2013; Calvillo, 2022; Montanari et al., 2024).

## Data Availability

The raw data supporting the conclusions of this article will be made available by the authors, without undue reservation.
