# Supplementary material for: Twenty years of health monitoring in a conventional neuroscience animal facility: challenges, strategies, and 3Rs-oriented approaches to animal welfare, personnel health, and research integrity
Source: Front Physiol. 2026 Jul 15;17:1821583. doi: 10.3389/fphys.2026.1821583 (PMC13416960; doi:10.3389/fphys.2026.1821583)
Supplement: Supplementary file 1 [file DataSheet1.pdf]

Supplementary Table S1. Pathogens included in the health-monitoring panels for mice and rats

Category	Pathogen / agent tested	Species	Monitoring frequency	Diagnostic method
Viruses	Mouse hepatitis virus (MHV)	Mouse only	Quarterly	ELISA / serology
	Mouse rotavirus / EDIM	Mouse only	Quarterly	ELISA / serology
	Minute virus of mice (MVM)	Mouse only	Quarterly	ELISA / serology
	Mouse parvovirus / MPV NS1	Mouse only	Quarterly	ELISA / serology
	Kilham rat virus (KRV)	Rat only	Quarterly	ELISA / serology
	Rat parvovirus (RPV)	Rat only	Quarterly	ELISA / serology
	Toolan's H-1 parvovirus	Rat only	Quarterly	ELISA / serology
	Pneumonia virus of mice (PVM)	Mouse and rat	Quarterly	ELISA / serology
	Sendai virus	Mouse and rat	Quarterly	ELISA / serology
	Theiler's encephalomyelitis virus / GDVII / TMEV	Mouse and rat	Quarterly	ELISA / serology
	Sialodacryoadenitis virus / Rat coronavirus (SDAV/RCV)	Rat only	Quarterly	ELISA / serology
	Reovirus type 3 (REO3)	Mouse and rat	Mouse: Quarterly; Rat: Annual	ELISA / serology
	Ectromelia virus / Mousepox	Mouse only	Annual	ELISA / serology
	Lymphocytic choriomeningitis virus (LCMV)	Mouse and rat	Annual	ELISA / serology
	Mouse adenovirus type 1 (MAV1, FL)	Mouse and rat	Annual	ELISA / serology
	Mouse adenovirus type 2 (MAV2, K87)	Mouse and rat	Annual	ELISA / serology

Category	Pathogen / agent tested	Species	Monitoring frequency	Diagnostic method
	Mouse cytomegalovirus (MCMV)	Mouse only	Annual	ELISA / serology
	Polyoma virus	Mouse only	Annual	ELISA / serology
	K virus	Mouse only	Annual	ELISA / serology
	Lactate dehydrogenase-elevating virus (LDV)	Mouse only	Annual	ELISA / serology
	Hantavirus / Korean hemorrhagic fever virus	Mouse and rat	Annual	ELISA / serology
	Mouse thymic virus (MTV)	Mouse only	Annual	ELISA / serology
	Murine norovirus	Mouse only	Annual	ELISA / serology
Bacteria, mycoplasmas and fungi	Citrobacter rodentium	Mouse only	Quarterly	Culture
	Clostridium piliforme / Tyzzer's disease	Mouse and rat	Quarterly	ELISA / serology
	Corynebacterium kutscheri	Mouse and rat	Quarterly	Culture
	Mycoplasma spp.	Mouse and rat	Quarterly	ELISA / serology
	Pasteurellaceae	Mouse and rat	Quarterly	Culture
	Salmonella spp.	Mouse and rat	Quarterly	Culture
	β-hemolytic streptococci	Mouse and rat	Quarterly	Culture
	Streptococcus pneumoniae	Mouse and rat	Quarterly	Culture
	Helicobacter spp.	Mouse and rat	Annual	PCR

Category	Pathogen / agent tested	Species	Monitoring frequency	Diagnostic method
	Streptobacillus moniliformis	Mouse and rat	Quarterly	Culture
	Bordetella bronchiseptica	Mouse and rat	Quarterly	Culture
	Leptospira spp.	Mouse and rat	Quarterly	Culture
	Staphylococcus aureus	Mouse and rat	Quarterly	Culture
	Pseudomonas aeruginosa	Mouse and rat	Quarterly	Culture
	Cilia-associated respiratory bacillus (CARB)	Mouse and rat	Annual	ELISA / serology
	Klebsiella pneumoniae / Klebsiella oxytoca	Mouse only	Quarterly	Culture
	Proteus spp.	Mouse and rat	Quarterly	Culture
	Pneumocystis carinii	Mouse and rat	Annual	PCR
Parasites and protozoa	Ectoparasites, species-level identification	Mouse and rat	Quarterly	Microscopic examination
	Endoparasites, species-level identification	Mouse and rat	Quarterly	Microscopic examination
	Trichomonas muris	Mouse and rat	Quarterly	Microscopic examination
	Entamoeba muris	Mouse and rat	Quarterly	Microscopic examination
	Syphacia spp.	Mouse and rat	Quarterly	Microscopic examination
	Chilomastix spp.	Mouse and rat	Quarterly	Microscopic examination
	Giardia muris	Mouse and rat	Quarterly	Microscopic examination

Category	Pathogen / agent tested	Species	Monitoring frequency	Diagnostic method
	**Spironucleus muris**	**Mouse and rat**	**Quarterly**	**Microscopic examination**
	**Encephalitozoon cuniculi**	**Mouse and rat**	**Annual**	**ELISA / serology**

Abbreviations: Quarterly = every 3 months; Annual = every 12 months; ELISA = enzyme-linked immunosorbent assay; PCR = polymerase chain reaction. Diagnostic methods are reported according to the available health-monitoring documentation: culture for most bacterial agents, ELISA/serology for viral agents and selected bacterial/mycoplasmal agents, PCR for Helicobacter spp. and Pneumocystis carinii, and microscopic examination for parasites and protozoa
